# Enhanced Photocatalytic Activity of Two-Dimensional Polar Monolayer SiTe for Water-Splitting via Strain Engineering

**DOI:** 10.3390/molecules28072971

**Published:** 2023-03-27

**Authors:** Di Gu, Wen Qin, Sumei Hu, Rong Li, Xingyuan Chen, Xiaoma Tao, Yifang Ouyang, Weiling Zhu

**Affiliations:** 1 Department of Physics, School of Science, Guangdong University of Petrochemical Technology, Maoming 525000, China; gudi@gdupt.edu.cn (D.G.);; 2 School of Physical Science and Technology, Guangxi University, Nanning 530004, China

**Keywords:** polar monolayer SiTe, photocatalytic water-splitting, strain engineering, first principle calculations

## Abstract

A two-dimensional (2D) polar monolayer with a polarization electric field can be used as a potential photocatalyst. In this work, first principle calculations were used to investigate the stability and photocatalytic properties of 2D polar monolayer SiTe as a potential promising catalyst in water-splitting. Our results show that the 2D polar monolayer SiTe possesses an indirect band gap of 2.41 eV, a polarization electric field from the (001) surface to the $\left(00\bar{1}\right)$ surface, a wide absorption region, and a suitable band alignment for photocatalytic water-splitting. We also discovered that the photocatalytic activity of 2D polar monolayer SiTe could be effectively tuned through strain engineering. Additionally, strain engineering, particularly compressive strain in the range from −1% to −3%, can enhance the photocatalytic activity of 2D polar monolayer SiTe. Overall, our findings suggest that 2D polar monolayer SiTe has the potential to be a promising catalyst for photocatalytic water-splitting using visible light.

## 1. Introduction

Growing global energy consumption and diminishing fossil fuel reserves have increased concerns about environmental pollution and energy shortages [1]. Renewable and clean energy sources, such as hydrogen [2,3], have become a strategic priority for sustainable development. Since Fujishima’s discovery in 1972 that hydrogen can be produced through photocatalysis using titanium dioxide (TiO_2_), scientists have assumed that this technology would aid the energy crisis [4]. It is an attractive method for producing hydrogen because it is clean, renewable, and abundant. The technological process of photocatalytic water-splitting requires only water, sunlight, and a catalyst and produces clean and renewable oxygen and hydrogen. The process involves the absorption of light by the photocatalyst, which generates electrons and holes. Therefore, it is considered a prospective technique for solving the pollution problem associated with the energy crisis. However, the efficiency of photocatalytic water-splitting is currently too low for utilization in industries [5,6].

To improve the efficiency of photocatalysis, researchers have focused on addressing the issue of carrier annihilation [7,8,9,10]. As we know, carrier annihilation results in the loss of charge carriers, which reduces the efficiency of the reaction. One way to address this problem is to use 2D materials. By reducing the distance that charge carriers need to migrate, the likelihood of carrier annihilation is decreased, and the efficiency of the reaction is improved. These 2D materials, such as g-C_3_N_4_ [11], group-III monochalcogenide [12], WS_2_ Nanosheet [13], MXene [14], and g-ZnO [15], were reported to have had high surface areas and short carrier diffusion lengths, which could effectively reduce the distance that charge carriers need to migrate and improve their efficiency [16,17,18,19,20].

In the recent literature, much research has suggested that 2D polar photocatalysts, which possess a polarization electric field, may be more effective at quickly separating photogenerated carriers [21,22,23,24]. There has been growing interest in 2D polar materials, such as group IV materials (GeS [25,26,27], GeSe [28], SiS [29,30], SiSe [31]), III_2_-VI_3_ group monolayer In_2_Se_3_ [32,33,34,35], monolayer Al_2_OS [36], and monolayer AgBiP_2_Se_6_, [37] as well as Janus monolayer materials (MoSSe [38,39,40], PtSSe [41,42], PtSO [43], and WSeTe [44]) for use as photocatalysts, thanks to the electric field that can aid in the separation of excited electron-hole pairs [45,46,47,48,49]. Two-dimensional polar monolayer SiM (M=S, Se and Te) possess high carrier mobility; therefore, they have been reported as potentially promising candidates for photocatalytic water-splitting [30,31,50], especially 2D polar monolayer SiTe, as it has a suitable band gap and absorbs visible light efficiently [50]. However, the effect of the polarization electric field on this material is not yet fully understood. Designing highly efficient catalysts based on 2D polar monolayer materials for photocatalytic water-splitting is critical in developing sustainable energy solutions.

Here, we used the first principles to systematically calculate the stability and photocatalytic properties of 2D polar monolayer SiTe. Our goal was to attain a better comprehension of the potential of this material for use in photocatalytic water-splitting and other applications. Our results show that 2D polar monolayer SiTe exhibits high dynamic, mechanical, and thermal stability. The band gap, band edge positions, and surface potential difference of 2D polar monolayer SiTe are suitable and helpful for water-splitting. Ultraviolet and visible light can be effectively absorbed by 2D polar monolayer SiTe. Furthermore, we found that the properties of 2D polar monolayer SiTe, including its band gap, surface potential difference, polarization electric field, absorption, and photocatalytic activity, can be tuned and enhanced by strain engineering. These findings suggest that 2D polar monolayer SiTe is a promising photocatalyst.

## 2. Results

**Figure 1:**
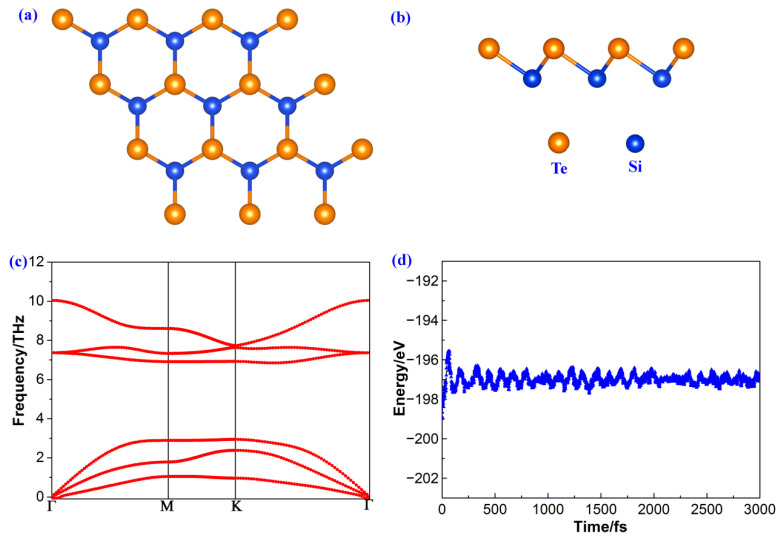
(**a**) Top view, (**b**) side view, (**c**) phonon dispersions, and (**d**) the total energy fluctuations during AIMD simulations of 2D polar monolayer SiTe.

Figure 1a shows the top view of the optimized hexagonal honeycomb structure of 2D polar monolayer SiTe, similar to graphene. Figure 1b shows the side view of 2D polar monolayer SiTe, which has a structure with two different layers: the top layer consists of Te atoms, and the bottom layer consists of Si atoms. Two-dimensional polar monolayer SiTe has an unsymmetrical structure along the vertical direction. To determine the most suitable lattice constant for 2D polar monolayer SiTe, we calculated the energy of different lattice constants and plotted the results in Figure S1. The results clearly show that when the lattice constant is 3.83 Å, the 2D polar monolayer SiTe has the lowest energy value, suggesting that it is the most suitable lattice of the monolayer structure. After a full relaxation, the values of the lattice constant, vertical layer distance, and bond distance were calculated, as presented in Table 1. The vertical layer distance is 1.53 Å, and the Si-Te bond distance is 2.69 Å, which are in accordance with previous literature [50,51,52,53].

To verify the dynamic stability of the 2D polar monolayer SiTe, we used the density functional perturbation theory method to calculate the phonon dispersion. This allowed us to identify any potential phonon instabilities that could indicate a lack of structural stability in the material. As shown clearly in Figure 1c, all the phonon dispersion modes of 2D polar monolayer SiTe are positive without any imaginary frequency, confirming that the structure of 2D polar monolayer SiTe is dynamically stable. In addition, a 5 × 5 × 1 supercell of 2D polar monolayer SiTe at a temperature of 300 K for a total time of 3000 fs with a time interval of 1 fs was set in the AIMD simulations. As Figure 1d shows, the total energy fluctuation during the dynamic simulations is comparatively small, further indicating the dynamic stability of 2D polar monolayer SiTe.

**Figure 2:**
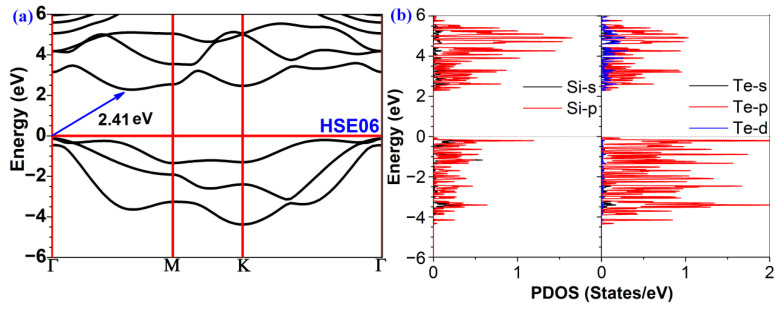
(**a**) The band structures and (**b**) the partial density of states (PDOS) of 2D polar monolayer SiTe.

The band structure is a significant factor that regulates the photocatalytic activity of a material. The band gap of a photocatalyst should be noteworthy. As shown in Figure 2a, our first principle calculations revealed that the valence band maximum (VBM) of 2D polar monolayer SiTe is at the *Γ* point. The conduction band minimum (CBM), on the other hand, is situated between the *Γ* and *M* points, indicating that 2D polar monolayer SiTe is an indirect semiconductor. Moreover, the band gap of 2D polar monolayer SiTe is 2.41 eV, calculated using the HSE06 functional. This result suggests that ultraviolet light and visible light can be efficiently absorbed by 2D polar monolayer SiTe. As shown in Figure 2b, the VBM of the partial density of states (PDOS) is below the Fermi energy level and is principally provided by the *p* orbitals of Te and Si atoms, whereas the CBM above the Fermi energy level is primarily provided by the *p* orbitals of Si atoms. The band gap shown in the PDOS is consistent with that shown in the band structure.

**Figure 3:**
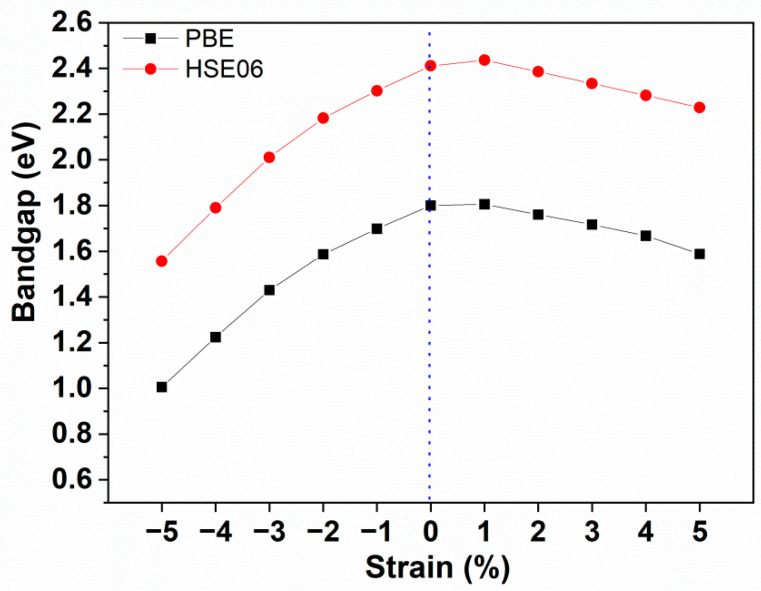
Band gap of 2D polar monolayer SiTe at different strains.

To tune the band structure properties of 2D polar monolayer SiTe effectively, the strain engineering method [54,55,56,57,58,59,60] is adopted for the structure of 2D polar monolayer SiTe by controlling the lattice constant of the primitive cell. As shown in Figure S2 and Figure 3, we applied in-plane biaxial strain to 2D polar monolayer SiTe ranging from −5% to +5% in steps of 1%. Here, a negative sign (−) is used to describe the compressive strain. In contrast, a positive sign (+) is used to describe the tensile strain. As we can see from Figure S2, the band structure of 2D polar monolayer SiTe can be effectively tuned by strain engineering. When a tensile strain from −1% to −5% is applied, the VBM is at the *Γ* point, and the CBM is situated between the *Γ* and *M* points, similar to the band structure without strain engineering. However, when the tensile strain is in the range from +1% to +5%, the VBM is located between the *K* and *Γ* points. As shown in Figure 3, the band gap of 2D polar monolayer SiTe, calculated by the HSE06 method, decreases from 2.41 eV to 1.56 eV as the compressive strain increases from 0 to −5%. For tensile strain, when the strain is +1%, the band gap is 2.44 eV, which is similar to the value without strain engineering. As the strain increases from +1% to +5%, the band gap decreases from 2.44 eV to 2.23 eV. These results demonstrate that strain engineering would be a feasible technique for controlling and tuning the band structure of 2D polar monolayer SiTe and increasing its activity to absorb solar energy in the ultraviolet and visible light range, which may well strengthen its photocatalytic activity.

**Figure 4:**
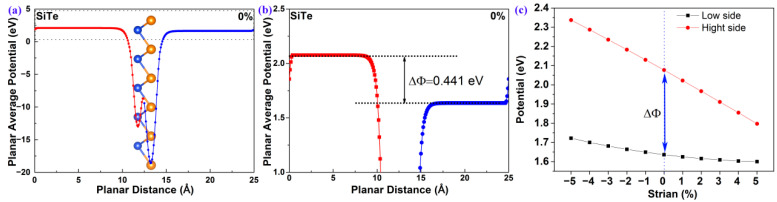
(**a**) The planar average potential of 2D polar monolayer SiTe; (**b**) an enlarged section of (**a**); (**c**) the surface potential difference (ΔΦ) of 2D polar monolayer SiTe at different strains.

As previously mentioned, 2D polar monolayer SiTe has an unsymmetrical structure along the vertical direction, with a bottom layer of Si atoms and a top layer of Te atoms. As shown in Figure 4a, the difference in electronegativity between Si and Te atoms results in an unsymmetrical planar average potential along the vertical direction. More importantly, the potential of the bottom surface $\left(00\bar{1}\right)$ is not equal to that of the top surface (001). According to the theory developed by the Yang group [22], the following equation can be employed to describe the relationship between both the surface potential difference (ΔΦ) and the polarization electric field ($E_{eff}$) [22].

(1) $$\Delta \Phi =eE_{eff}d$$

Here, *e* is the elementary charge constant, and *d* is the distance between the Si atomic surface and Te atomic surface.

The larger ΔΦ, the larger $E_{eff}$. As shown in Figure 4b, the ΔΦ, calculated by the surface vacuum energy difference between the Si atomic surface and the Te atomic surface, is 0.441 eV. This indicates that 2D polar monolayer SiTe possesses a polarization electric field $E_{eff}$, and the direction of the polarization electric field is from the (001) surface to the $\left(00\bar{1}\right)$ surface. As we know, the carriers can be separated quickly and irreversibly under the polarization electric field. Therefore the $E_{eff}$ and the ΔΦ in 2D polar monolayer SiTe are useful for improving the performance of photocatalytic water-splitting. Additionally, the photocatalyst’s band gap $E_{G}$, which is required for photocatalytic water-splitting, can be reduced under the effect of the surface potential difference (ΔΦ) according to the following equation [22],

(2) $$E_{G}>1.23-\Delta \Phi$$

These photocatalysts, such as 2D polar monolayer SiTe, can absorb more solar energy in the infrared and visible region for photocatalytic water-splitting. Therefore, tuning the surface potential difference ΔΦ would be an effective and useful way to control photocatalytic activity.

As Figure S3 shows, the planar average potential of 2D polar monolayer SiTe can be regularly controlled by strain engineering. The surface potential difference ΔΦ increases under the effect of compressive strain and decreases under the effect of tensile strain. The surface potential difference ΔΦ decreases from 0.615 eV to 0.197 eV as the strain engineering increases monotonically from −5% to +5%. Figure 4c shows the changing trends of the surface potential difference ΔΦ and the surface vacuum energy ($E_{vac}$) at different strains. The $E_{vac}$ of both the Si and Te atomic surfaces decreases monotonically with increasing strain engineering from −5% to +5%. Moreover, the $E_{vac}$ decrease trend of the Si atomic surface is obvious, but the $E_{vac}$ decrease trend of the Te atomic surface is slight. The results indicate that the ΔΦ of 2D polar monolayer SiTe can be effectively tuned via strain engineering.

**Figure 5:**
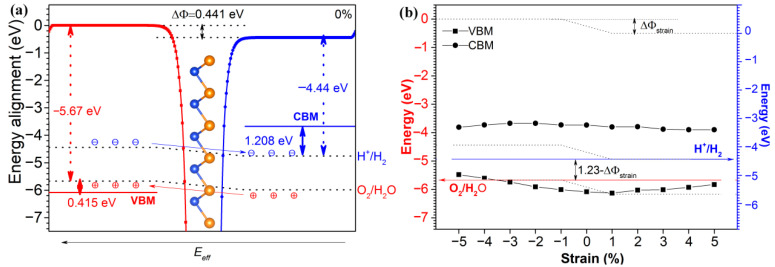
(**a**) The band alignment of 2D polar monolayer SiTe; (**b**) the Band alignment as a function of strain engineering.

To appraise the suitability of 2D polar monolayer SiTe for photocatalytic water-splitting, we calculated its band alignment using the method described in previous studies [21,22]. The band alignment of a material is a critical factor in determining its suitability for photocatalytic water-splitting. For a potential photocatalyst to be effective, the energy level of VBM and CBM must be suitable. As we can see clearly from Figure 5a, the band alignment of 2D polar monolayer SiTe was calculated by the method reported in the previous literature [22,31]. Firstly, the VBM was calculated by,

(3) $$E_{VBM}=\varphi (\infty )-E_{F}$$

Here, $E_{VBM}$ is presumed to be the work function throughout this situation. φ(∞) and $E_{F}$, respectively, stand in for the Fermi energy level and the vacuum electrostatic potential. Secondly, the CBM was obtained by,

(4) $$E_{CBM}=E_{CBM}+E_{g}$$

Here, $E_{g}$ is the band gap shown in Figure 2a.

As shown in Figure 5a, with the vacuum energy level as the reference substance, the reduction potential (H^+^/H_2_) and the oxidation potential (H_2_O/O_2_) are −4.44 eV and −5.67 eV, respectively. Since the ΔΦ is 0.441 eV in 2D polar monolayer SiTe, the energy level of the (001) surface is not equal to that of the $\left(00\bar{1}\right)$ surface. The direction of the polarization electric field $E_{eff}$ is from the (001) surface to the $\left(00\bar{1}\right)$ surface. Therefore electrons and holes are moved to the (001) surface and the $\left(00\bar{1}\right)$ surface, respectively. On the (001) surface, the H_2_O is reducesed by the electrons according to the following equation,

(5) $$4e^{-}+4H_{2}O\to 2H_{2}+4OH^{-}$$

In contrast, on the $\left(00\bar{1}\right)$ surface, the H_2_O is oxidized the holes according to the following equation,

(6) $$4h^{+}+2H_{2}O\to O_{2}+4H^{+}$$

The CBM of 2D polar monolayer SiTe is 1.208 eV higher than the energy level of H^+^/H_2_ (−4.44 eV), and the VBM is 0.415 eV lower than the energy level of H_2_O/O_2_ (−5.67 eV). Moreover, under the effect of an electric field $E_{eff}$, the electrons and holes could be separated quickly; therefore, oxidation and reduction reactions would be carried out efficiently. The results indicated that the band alignment of 2D polar monolayer SiTe is ideal for photocatalytic water-splitting, which involves the production of hydrogen through the use of sunlight and a photocatalyst.

As we can see from Figure S4 and Figure 5b, the CBM of 2D polar monolayer SiTe with strain engineering ranging from −5% to +5% is higher than the reduction potential level. Compared to the oxidation potential level, the VBM is lower, with strain engineering ranging from −3% to +5%. However, at −5% and −4% compressive strain, the VBM is higher than the magnitude of oxidation potential. This demonstrates that the band alignment of 2D polar monolayer SiTe continues to be well tailored towards photocatalytic water-splitting when strain engineering is applied within the range from −3% to +5%.

The Gibbs free energy difference (Δ*G*) in the hydrogen reduction was determined using the following equation to investigate the dependence power of total water-splitting [61],

(7) $$\Delta G=\Delta E+\Delta E_{zpe}-T\Delta S$$

Here, Δ*E* is the adsorption energy, Δ*E_zpe_* is the difference in zero-point energy, *T* is the system temperature (298.15 K, in this work), and Δ*S* is the entropy difference.

**Figure 6:**
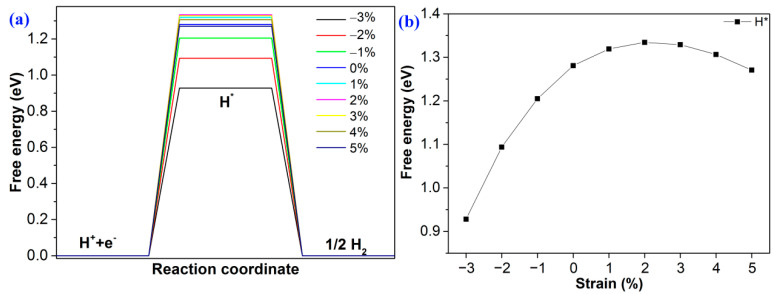
(**a**) The Gibbs free energy changes of HER at different strains; (**b**) the trend of Δ*G* at different strains.

As shown in Figure 6a, the value of Δ*G* is 1.28 eV. To control and decrease the value of ΔG, strain engineering is adopted to tune the value. As shown in Figure 6b, the Δ*G* decreases from 1.24 eV to 0.92 eV as the compressive strain decreases from 0 to −3%. For tensile strain, when the strain is +2%, the Δ*G* is 1.33 eV. As the strain increases from +2% to +5%, the band gap decreases from 1.33 eV to 1.27 eV regularly. Notably, the value of Δ*G* can be effectively tuned via strain engineering.

**Figure 7:**
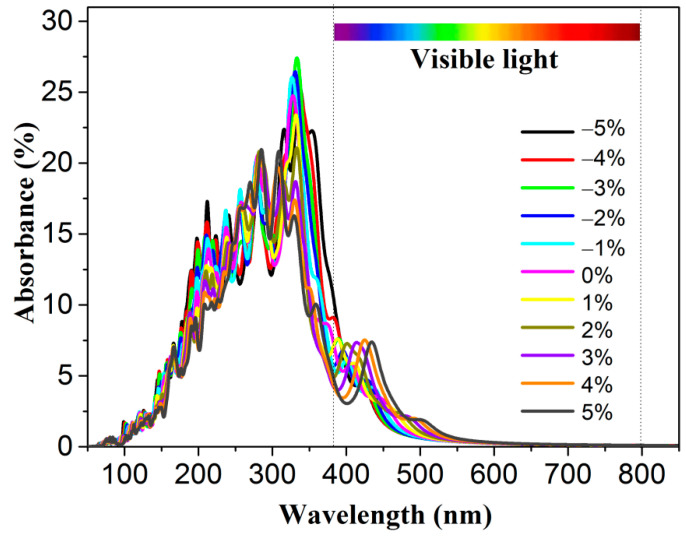
The absorbance of 2D polar monolayer SiTe under strain engineering.

The following equation was used to determine the absorption coefficient α(ω) of 2D polar monolayer SiTe:

(8) α(ω)=2ω((ε1(ω))2+(ε2(ω))2−ε1(ω))12

where $\varepsilon_{1}(\omega )$ and $\varepsilon_{2}(\omega )$, respectively, represent the real and imaginary components of the dielectric function. As seen from Figure S5a, when the energy is less than 2.00 eV, the absorbance is almost zero. In contrast, when the energy is about 2.50 eV, the absorbance increases dramatically as the energy increases, indicating that some visible light is absorbed efficiently by the 2D polar monolayer SiTe. Moreover, the absorption edge was obtained using the Tauc plot method [62]. As shown in Figure S5b, the band gap *E_g,_* obtained by the optical properties is 2.38 eV, similar to the band gap calculated by the band structures using the HSE06 method. This confirms that the solar light, which has energy larger than the band gap, will be absorbed efficiently by the 2D polar monolayer SiTe. This indicates that 2D polar monolayer SiTe has the potential to be an efficient photocatalyst with high solar energy conversion efficiency. The band gap of 2D polar monolayer SiTe, which is approximately 2.41 eV, plays a role in improving solar energy conversion efficiency. As shown in Figure 7, strain engineering can shift the absorption edge to longer wavelengths, increasing the absorption of visible light. This is due to the decrease in the band gap of 2D polar monolayer SiTe with increasing strain. The results suggest that strain engineering is a convenient and useful strategy for tuning the optical absorption properties of 2D polar monolayer SiTe.

## 3. Methods

We used the Vienna ab initio simulation package (VASP) [63] with the projector-augmented-wave (PAW) method [64] to calculate the electronic and structural properties of 2D polar monolayer SiTe. To calculate the exchange-correlation energy, the Perdew Burke Ernzerhof (PBE) functional of generalized gradient approximation (GGA) was adopted [65]. The DFT-D3 [66] was utilized to account for long-range van der Waals interactions [67,68]. The PBE method tends to underestimate the band gap, so we also used the HSE06 hybrid functional method [69] to obtain a more accurate band gap value. The plane-wave expansion of the wave function had an energy cutoff of 500 eV. We set the energy convergence and maximum Hellmann–Feynman force convergence criteria to 10^−6^ eV and 10^−3^ eV/Å, respectively. The first integration of the Brillouin zone was performed using the Gamma center method in KPOINTS [70]. Structural optimization and static calculations were performed using 12 × 12 × 1 and 15 × 15 × 1 grids, respectively. The vacuum region in the z direction had a thickness of 20 Å to prevent interactions from the periodic structure. Ab initio molecular dynamics (AIMD) simulations were calculated with the canonical ensemble method [71] to investigate the stability of crystal structure. A 5 × 5 × 1 supercell of 2D polar monolayer SiTe at a temperature of 300 K for a total time of 3000 fs with a time interval of 1 fs was set in the simulations. VASPKIT [72] software was applied to generate electronic data from the raw calculated data.

## 4. Conclusions

In conclusion, our study, which used first principle calculations, has shown that the 2D polar monolayer SiTe is a potential promising catalyst for water-splitting using visible light. According to the results, the 2D polar monolayer SiTe possesses an indirect band gap of 2.41 eV. The 2D polar monolayer SiTe can absorb ultraviolet and visible light in a wide range. What is more, in the 2D polar monolayer SiTe, the polarization electric field helps to reduce the likelihood of photoinduced electron–hole pair recombination and lower the band gap necessary for water-splitting. The direction of the polarization electric field is from the (001) surface to the $\left(00\bar{1}\right)$ surface. The band alignments of 2D polar monolayer SiTe are compatible with the redox potential, making it capable of producing hydrogen and oxygen. Moreover, the electronic, optical, and photocatalytic properties of the 2D polar monolayer SiTe can be controlled and tuned through strain engineering. Additionally, strain engineering, particularly compressive strain in the range from −1% to −3%, can enhance the photocatalytic activity of 2D polar monolayer SiTe. Overall, the suitable band gap, polarization electric field, and suitable band alignments strongly suggest that the 2D polar monolayer SiTe is a potentially promising, high-efficiency catalyst for photocatalytic water-splitting using visible light.

## Figures and Tables

**Table 1:** Comparison of the calculated values of lattice constant (*a*), vertical layer distance (*d*), bond distance (*l*), and band gap (*E_g_*).

	*a* (Å)	*d* (Å)	*l* (Å)	*E_g_* (eV)		References
				PBE	HSE06	
SiTe	**3.83**	**1.53**	**2.69**	**1.80**	**2.41**	This work
	3.83	1.53	2.69	1.83	2.43	[50]
	3.83	1.53	2.69	1.83	2.36	[51]
	3.83	-	-	1.83	2.49	[52]
	3.835	-	2.690	1.833	-	[53]

## Data Availability

The data that support the findings of this study are available from the corresponding author upon reasonable request.

## Supplementary Materials

The following supporting information can be downloaded at: https://www.mdpi.com/article/10.3390/molecules28072971/s1, Figure S1: The energy as a function of lattice constant of monolayer SiTe; Figure S2: The band structures of monolayer SiTe under strain engineering; Figure S3: The planar average potential of monolayer SiTe under strain engineering; Figure S4: The band alignment of monolayer SiTe under strain engineering; Figure S5: The Absorbance and Absorption edge of 2D polar monolayer SiTe.
