# Supplementary material for: Enhanced Photocatalytic Activity of Two-Dimensional Polar Monolayer SiTe for Water-Splitting via Strain Engineering
Source: Molecules. 2023 Mar 27;28(7):2971. doi: 10.3390/molecules28072971 (PMC10096314; doi:10.3390/molecules28072971)
Supplement: Supplementary file 1 [file molecules-28-02971-s001.zip › molecules-2269153-supplementary.pdf]

Supporting Information

Enhanced photocatalytic performance of Two-dimensional Polar Monolayer SiTe for water splitting via strain engineering

Di Gu [1], Wen Qin [1], Sumei Hu [1], Rong Li [1], Xingyuan Chen [1,*], Xiaoma Tao [2], Yifang Ouyang [2] and Weiling Zhu [1,*]

[1] Department of Physics, School of Science, Guangdong University of Petrochemical Technology, Maoming 525000, China; gudi@gdupt.edu.cn (D.G.)

[2] School of Physical Science and Technology, Guangxi University, Nanning 530004, China

* Correspondence : chenxingyuan@gdupt.edu.cn (X.C.); zhuweiling@gdupt.edu.cn (W.Z.)

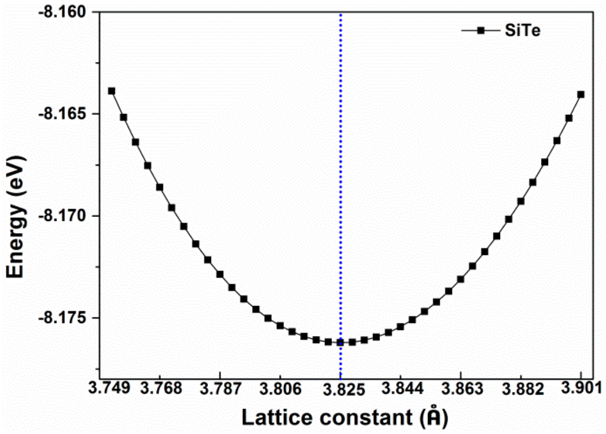

Figure S1. The energy as a function of lattice constant of monolayer SiTe.

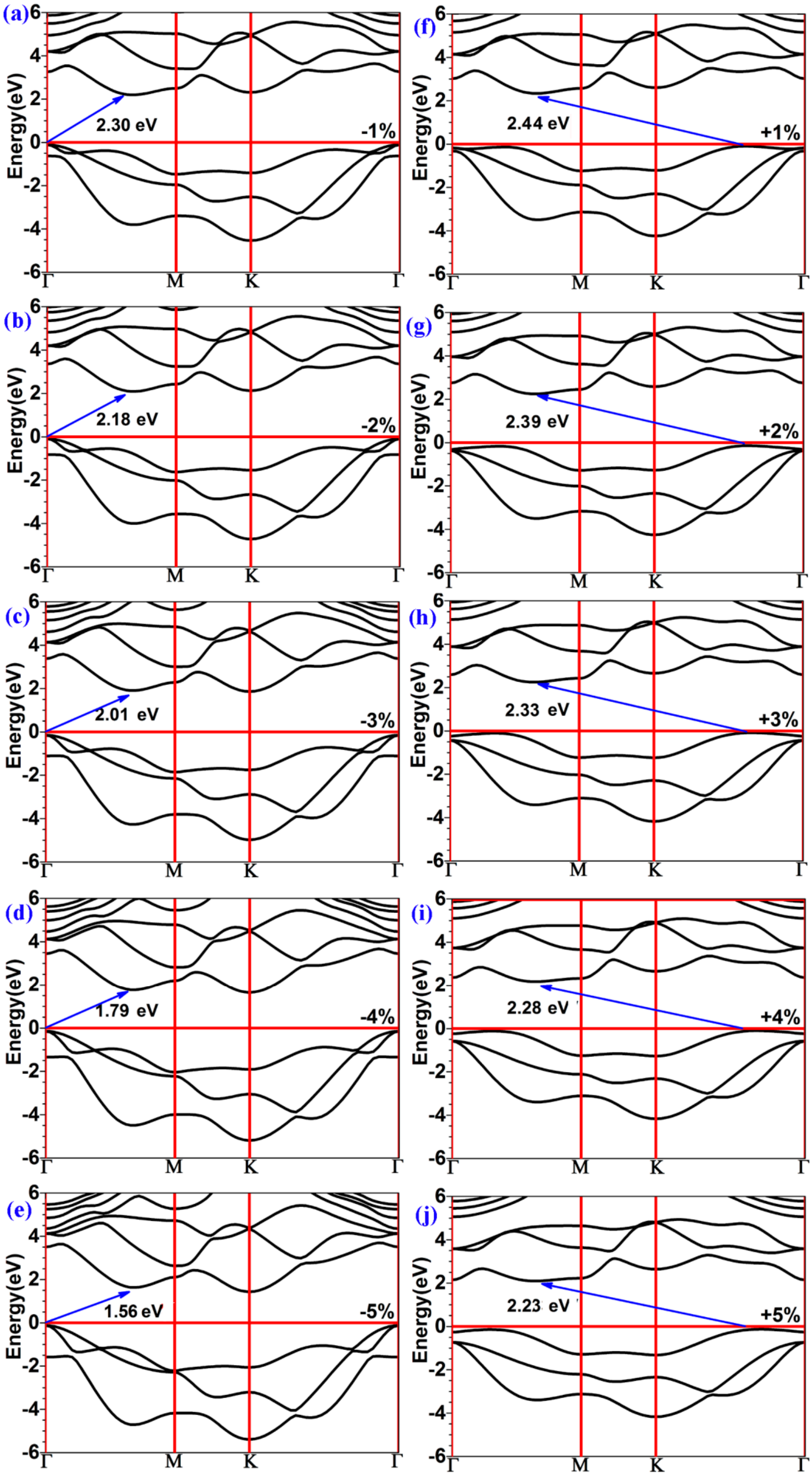

Figure S2. The band structures of monolayer SiTe under strain engineering

(a) -1%, (b) -2%, (c) -3%, (d) -4%, (e) -5%, (f) +1%, (g) +2%, (h) +3%, (i) +4%, (j) +5%

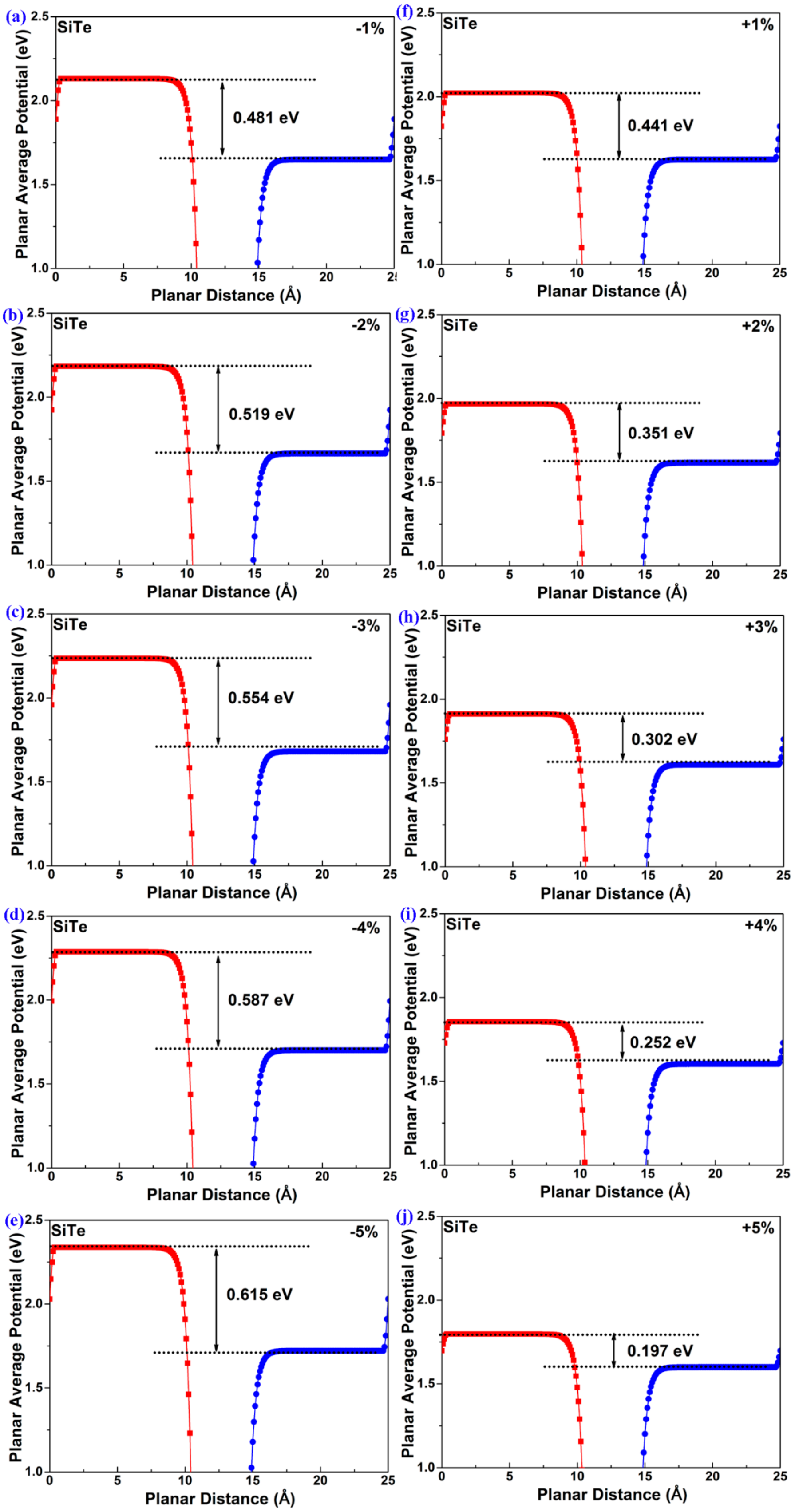

Figure S3. The planar average potential of monolayer SiTe under strain engineering (a) -1%, (b) -2%, (c) -3%, (d) -4%, (e) -5%, (f) +1%, (g) +2%, (h) +3%, (i) +4%, (j) +5%

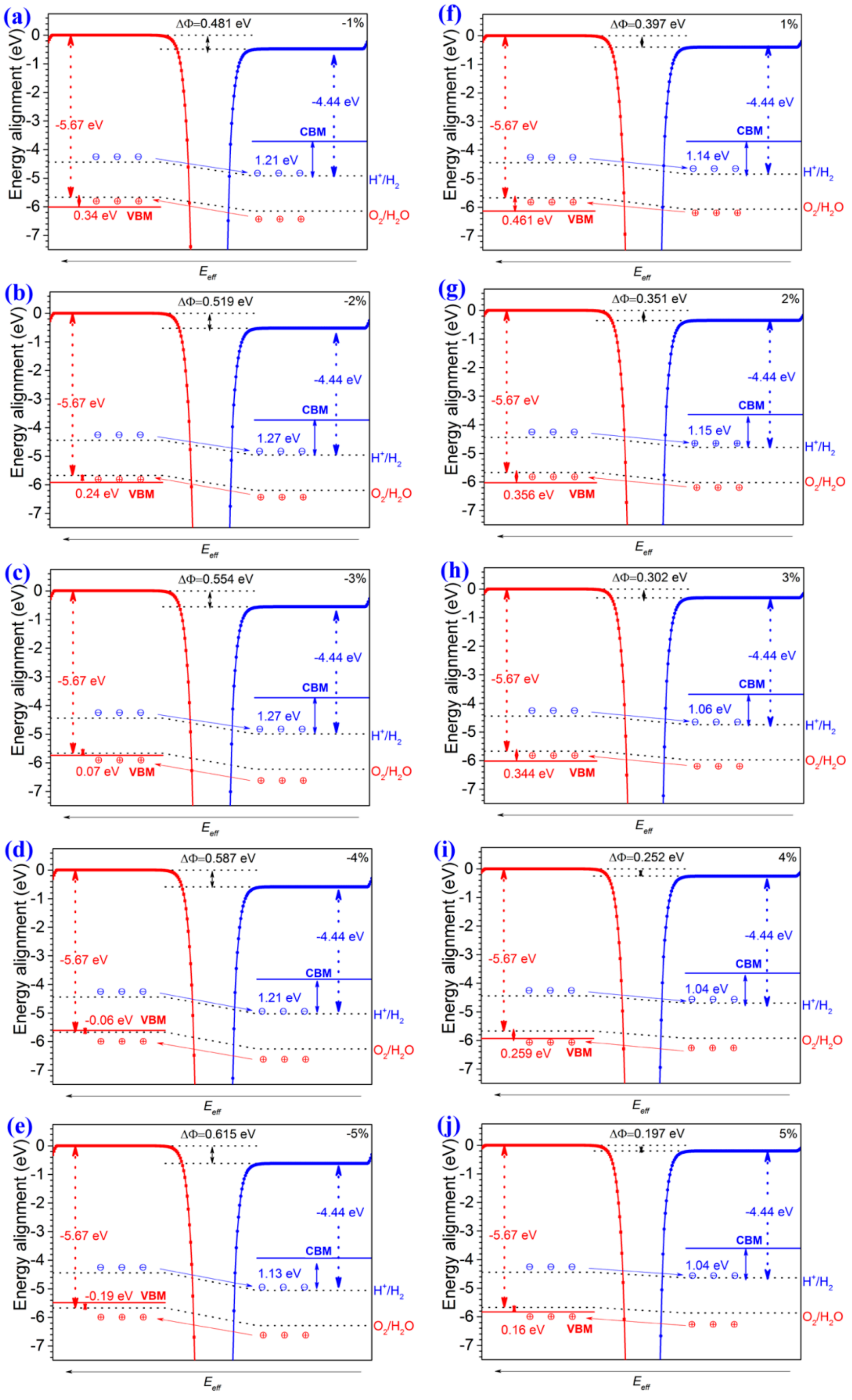

Figure S4. The band alignment of monolayer SiTe under strain engineering

(a) -1%, (b) -2%, (c) -3%, (d) -4%, (e) -5%, (f) +1%, (g) +2%, (h) +3%, (i) +4%, (j) +5%

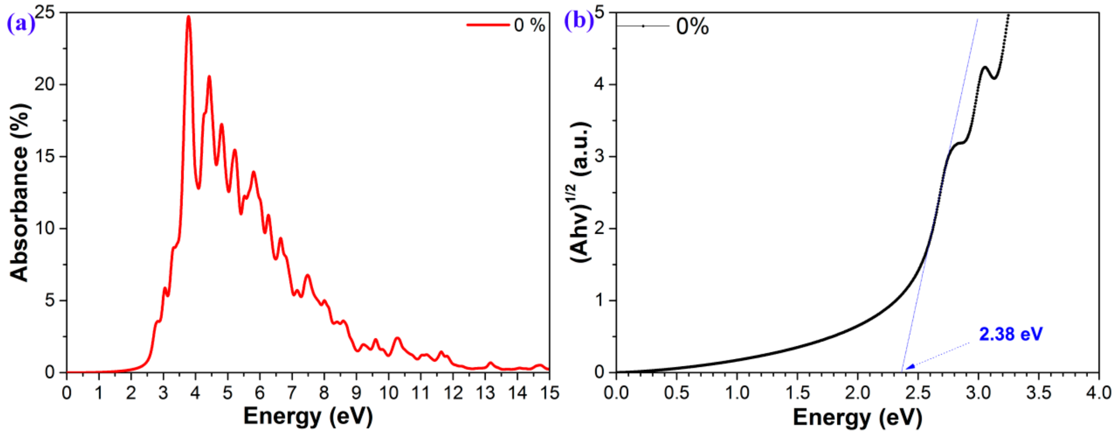

Figure S5. (a) The Absorbance and (b) Absorption edge of 2D polar monolayer SiTe.
